# Epithelial-Mesenchymal Transition Induces GSDME Transcriptional Activation for Inflammatory Pyroptosis

**DOI:** 10.3389/fcell.2021.781365

**Published:** 2021-11-26

**Authors:** Chenqiang Jia, Zhuqing Zhang, Jun Tang, Mei-Chun Cai, Jingyu Zang, Kaixuan Shi, Yunheng Sun, Jie Wu, Hailei Shi, Weiping Shi, Pengfei Ma, Xiaojing Zhao, Zhuang Yu, Yujie Fu, Guanglei Zhuang

**Affiliations:** ^1^ State Key Laboratory of Oncogenes and Related Genes, Shanghai Cancer Institute, Ren Ji Hospital, Shanghai Jiao Tong University School of Medicine, Shanghai, China; ^2^ School of Biomedical Engineering and Med-X Research Institute, Shanghai Jiao Tong University, Shanghai, China; ^3^ Shanghai Key Laboratory of Gynecologic Oncology, Ren Ji Hospital, Shanghai Jiao Tong University School of Medicine, Shanghai, China; ^4^ Department of Thoracic Surgery, Ren Ji Hospital, Shanghai Jiao Tong University School of Medicine, Shanghai, China; ^5^ Department of Pathology, The Affiliated Hospital of Qingdao University, Qingdao, China; ^6^ Department of Oncology, The Affiliated Hospital of Qingdao University, Qingdao, China

**Keywords:** epithelial-mesenchymal transition, GSDME, transcription activation, pyroptosis, ZEB1/2

## Abstract

GSDME is a newly recognized executor of cellular pyroptosis, and has been recently implicated in tumor growth and immunity. However, knowledge about the molecular regulators underlying GSDME abundance remains limited. Here, we performed integrative bioinformatics analyses and identified that epithelial-mesenchymal transition (EMT) gene signatures exhibited positive correlation with GSDME levels across human cancers. A causal role was supported by the observation that EMT dictated GSDME reversible upregulation in multiple experimental models. Mechanistically, transcriptional activation of *GSDME* was directly driven by core EMT-activating transcription factors ZEB1/2, which bound to the *GSDME* promoter region. Of functional importance, elevated GSDME in mesenchymally transdifferentiated derivatives underwent proteolytic cleavage upon antineoplastic drug exposure, leading to pyroptotic cell death and consequent cytokine release. Taken together, our findings pinpointed a key transcriptional machinery controlling GSDME expression and indicated potential therapeutic avenues to exploit GSDME-mediated inflammatory pyroptosis for the treatment of mesenchymal malignancies.

## Introduction

Pyroptosis is a lytic type of programmed cell death characterized by cellular swelling and pore formation on cytomembrane, resulting in passive release of intracellular contents (He et al., 2015; Kayagaki et al., 2015; Shi et al., 2015; Liu et al., 2016; Strasser and Vaux, 2020). Several members of the gasdermin family (GSDMA, GSDMB, GSDMC, GSDMD, GSDME, and PJVK) have been recently identified to exert pyroptotic activity under different circumstances (Ding et al., 2016; Broz et al., 2020). For instance, GSDME-dependent pyroptosis can be triggered by chemotherapy or molecular targeted drugs in malignant cells (Rogers et al., 2017; Wang et al., 2017; Lu et al., 2018). During this process, GSDME is cleaved by activated caspase-3 to execute membrane permeabilization and proinflammatory cell killing. Indeed, antitumor immune responses during small-molecule therapy are reportedly enhanced by GSDME-mediated pyroptosis, which causes extracellular outflow of immunostimulatory factors and subsequent T cell propagation (Erkes et al., 2020; Rosenbaum et al., 2021; Zhang et al., 2021). Additionally, granzyme B from cytotoxic lymphocytes, by directly cleaving GSDME, induces caspase-independent pyroptosis and reinforces antineoplastic immunity (Zhang et al., 2020). Therefore, GSDME plays dual tumor-suppressive roles and lies in the intersection between pyroptotic cell death and rejuvenation of immune microenvironment, offering the possibility of harnessing its function to treat cancer.

However, tumor cells may delicately adopt various strategies to escape from GSDME-elicited suppression. Previous studies have extensively documented a molecular machinery involving epigenetic inactivation due to *GSDME* promoter hypermethylation in a range of human cancers (Akino et al., 2007; Kim et al., 2008a; Kim et al., 2008b; Yokomizo et al., 2012; Wang et al., 2013; Ibrahim et al., 2019; De Schutter et al., 2020). As a result, DNA methyltransferase inhibitors restore *GSDME* gene transcription and reinstate chemotherapy sensitivity (Wang et al., 2017; Fan et al., 2019). Nonetheless, we and others have observed ubiquitous expression of GSDME across multiple malignancies (Lu et al., 2018; Wu et al., 2019; Yu et al., 2019), suggesting that alternative regulatory mechanisms probably exist in certain context. Further understanding the determinants of GSDME levels holds the promise to therapeutically exploit disparate cell death modalities and deliberately produce pyroptosis-associated cytotoxicity and immune stimulation for cancer control.

In this study, we demonstrate that in addition to promoter methylation, epithelial-mesenchymal transition (EMT) dictates *GSDME* gene expression in diverse tumor models. We provide evidence that core EMT-activating transcription factors (EMT-TFs) ZEB1/2 are responsible for *GSDME* upregulation. Importantly, increased GSDME in mesenchymal cancer cells is functional to execute therapy-induced pyroptosis, leading to plasma membrane leakage and proinflammatory cytokine release.

## Materials and Methods

### Cell Culture and Reagents

All cell lines were obtained from ATCC, where cell characterization was verified via polymorphic short tandem repeat (STR) profiling. Cells were cultured in RPMI1640 (Invitrogen) supplemented with 10% fetal bovine serum (Gibco), 1% GlutaMAX, and 1% sodium pyruvate solution. TGFβ was purchased from Cell Signaling Technology, and used to treat cells at a final concentration of 5 nM. Decitabine, thioguanine, osimertinib, erlotinib, and salinomycin were purchased from Selleck Chemicals. All inhibitors were reconstituted in DMSO (Sigma-Aldrich) at a stock concentration of 10 mM, and used to treat cancer cells at a final concentration of 4 μM. Lactate dehydrogenase (LDH) assays were performed using CytoTox 96 Non-Radioactive Cytotoxicity Assay Kit (Promega).

### Plasmids, sgRNA and Virus Infection

Plasmids for gene overexpression were generated using the Gateway Cloning System (Invitrogen). All gene coding sequences were amplified using the PrimeSTAR GXL DNA Polymerase Kit (Takara) and authenticated by Sanger sequencing. The CRISPR-Cas9 system was applied to knock out genes of interest. The sequences of sgRNAs were provided in Supplementary Table S1. For virus packaging, 5 μg of lentiviral constructs, 5 μg of Δ8.9 plasmid, and 3 μg of VSVG plasmid were transfected into HEK293T cells with Lipofectamine 2000 (Invitrogen). Virus-containing supernatant was gathered 48 h after transfection and mixed with 8 μg/ml polybrene to infect target cells. Infected cells were selected with 5 μg/ml puromycin or blasticidin.

### Western Blot Analysis

Cells were lysed in RIPA buffer (50 mM Tris pH 7.4, 150 mM NaCl, 1% NP-40, 0.1% SDS, and 2 μM EDTA) supplemented with proteinase inhibitors (Roche). The cell lysates (20 μg) were subjected to SDS-PAGE and Western blot. Antibodies for the following proteins were used: E-cadherin (#3195), SLUG (#9585), SNAIL (#3879), Vimentin (#5741), TWIST (#46702), ZEB2 (#97885), and GAPDH (#8884, Cell Signaling Technology); ZEB1 (#21544-1-AP, Proteintech); GSDME (ab215191, Abcam).

### RNA Extraction and Quantitative PCR

Total RNA from cells was extracted using Trizol (Invitrogen) and subjected to reverse transcription with the HiScript III Q RT SuperMix Kit (Vazyme). The quantitative PCR (qPCR) was conducted on the Applied Biosystems ViiA7 machine. Relative mRNA expression of each gene was normalized to GAPDH as the endogenous control. At least three biological replicates were included for each condition.

### Luciferase Reporter Assay

The promoter region of human GSDME was cloned into the luciferase reporter vector (GeneCopoeia). HEK293T cells were transfected in 12-well plates with Lipofectamine 2000 (Invitrogen). Forty-eight hours after transfection, luciferase assays were conducted using Secrete-Pair Dual Luminescence Assay Kit (GeneCopoeia) following the manufacturer’s instructions. The gaussia luciferase activity was normalized to secreted alkaline phosphatase expression control. The normalized value was then normalized to the detection value of the control construct. Means and standard deviations were calculated in three independent replicates for each condition.

### Chromatin Immunoprecipitation

Cells were crosslinked with RPM1640 medium supplemented with 1% formaldehyde for 10 min at room temperature, and terminated with 1/20 volume of 2.5 M glycine. The cells were collected and washed by cold PBS. Cell samples were suspended and rocked in three lysis buffers in sequence as previously described (Lee et al., 2006). The lysates were sonicated with an ultrasonic processor VC505 (Sonics and Materials) for obtaining 200–500 bp fragments. Subsequently, 50 μl lysates were reserved as whole cell extract (WCE). The remaining lysates were incubated with antibody-conjugated magnetic beads overnight. For preparing beads, 100 μl of magnetic beads (Invitrogen) were incubated with 10 μg ZEB1 (#21544-1-AP, Proteintech), normal rabbit IgG (#2729, Cell Signaling Technology) or ZEB2 (sc-271984, Santacruz) antibodies overnight, respectively. The fragments-attached beads were washed several times, and eluted by elution buffer (50 mmol/L Tris-HCl pH 8.0, 10 mmol/L EDTA, 1% SDS) at 65°C for 15 min. Both the elution samples and WCE samples were incubated overnight at 65°C for crosslink reversal. These samples were treated with RNase A and Proteinase K, and then purified with QIAquick PCR Purification Kit (Qiagen). Purified DNA samples were employed to confirm the enrichment of target DNA fragments by quantitative PCR. The enrichment of target sequences in samples from specific immunoprecipitation was normalized to their amplification in the sample from IgG immunoprecipitation.

### Luminex Liquid Suspension Chip Detection

The Bio-Plex Pro Human Cytokine 27-plex assay (Bio-Rad) was performed for quantifying released cytokines using Luminex liquid suspension chip detection technology according to the manufacturer’s instructions. Briefly, culture supernatants were collected from cells treated with erlotinib for 72 h, and incubated with specific capture beads. Subsequently, detection antibodies and Phycoerythrin (PE)-conjugated streptavidin were added into each well in sequence. The fluorescent signal analysis was carried out with a Bio-Plex MAGPIX Multiplex Reader.

### Data Extraction and Preprocessing

The RNA sequencing data of 33 TCGA cancer types were provided by the Pan-Cancer Atlas (Hoadley et al., 2018; Liu et al., 2018; Sanchez-Vega et al., 2018) and downloaded from the UCSC Xena Explorer (cohort: TCGA Pan-Cancer). The transcriptomic data of the gasdermin family and EMT transcription factors in cancer cell lines were obtained from Broad Institute Cancer Cell Line Encyclopedia (CCLE) (Ghandi et al., 2019). All datasets were processed as described previously (Cai et al., 2019). The methylation data were obtained from TCGA Pan-Cancer Atlas and CCLE, and then analyzed as described previously (Du et al., 2010; Ghandi et al., 2019). The β average value of all CpG sites between TSS −2000–+1000 was identified as the methylation value of the indicated gene in each sample. The methylation statuses of promoters were assessed as hypomethylation, partial methylation, and hypermethylation as described previously (Maekawa et al., 2019).

### Single-Sample Gene Set Enrichment Analysis

The gene signatures were derived from the Molecular Signatures Database (MSigDB) (Subramanian et al., 2005). We quantified the relative level of each gene signature in each sample by performing the single-sample gene set enrichment analysis (ssGSEA). The GSVA package in R was employed to calculate ssGSEA scores (Hänzelmann et al., 2013).

### Statistical Analysis

Statistical analysis was performed using the GraphPad Prism software. In all experiments, comparisons between two groups were based on two-sided Student’s t-test. P-values of <0.05 were considered statistically significant. The Pearson correlation analysis was used to evaluate the correlation between continuous factors. The geneset network was constructed using the Cytoscape software.

## Results

### Positive Correlation of Expression Levels Between GSDME Gene and Epithelial-Mesenchymal Transition Markers in Human Cancer

**FIGURE 1:**
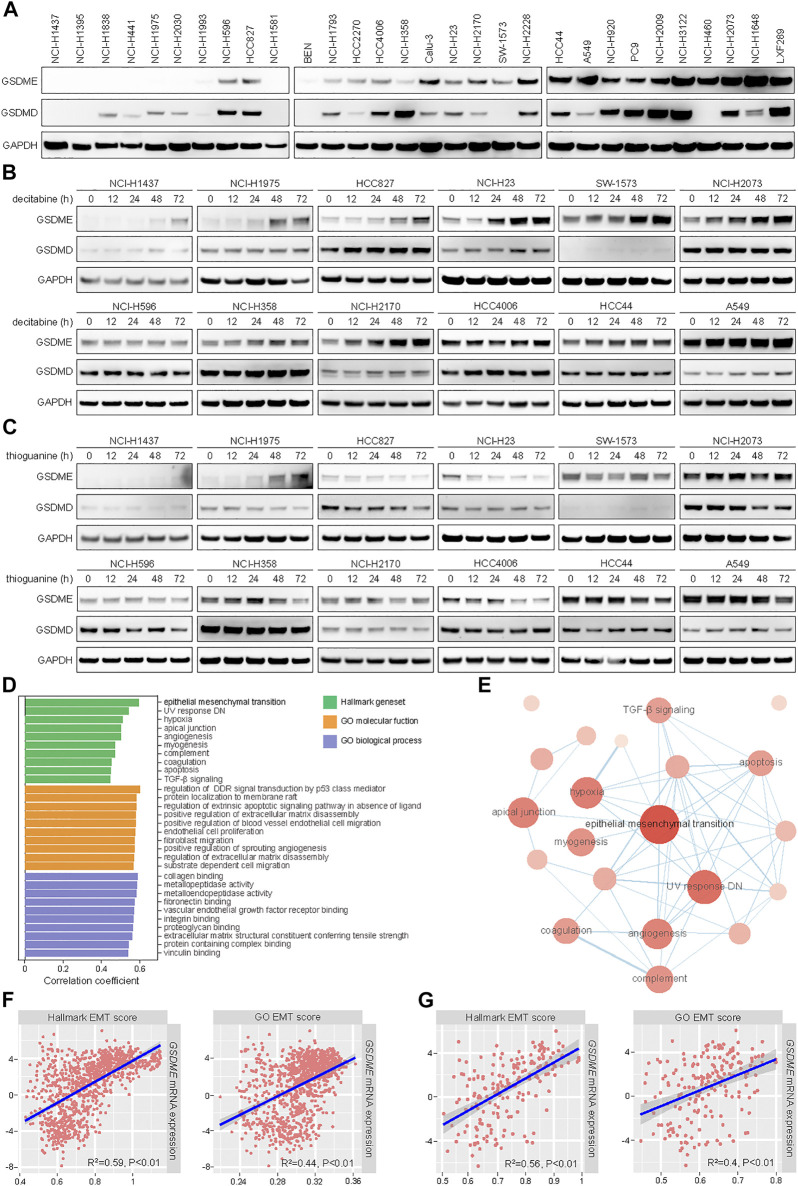
Positive correlation of expression levels between *GSDME* gene and EMT markers in human cancer. **(A)** The protein expression of GSDME and GSDMD was analyzed by Western blot in 30 lung cancer cell lines. **(B)** Indicated lung cancer cell lines with variable GSDME levels were treated with decitabine (4 μM) at a time course manner and analyzed by Western blot for GSDME and GSDMD protein expression. **(C)** Indicated lung cancer cell lines with variable GSDME levels were treated with thioguanine (4 μM) at a time course manner and analyzed by Western blot for GSDME and GSDMD protein expression. **(D)** Hallmark gene sets and gene ontology terms with ssGSEA scores that positively correlated with *GSDME* transcript expression in CCLE cancer cell lines. **(E)** The interaction network of hallmark gene sets with ssGSEA scores that positively correlated with *GSDME* transcript expression in CCLE cancer cell lines. **(F)** Scatterplots with linear regression line and shaded 95% confidence region for correlation estimation of *GSDME* transcript expression and EMT ssGSEA scores in CCLE cancer cell lines. R indicated Pearson correlation coefficient. **(G)** Scatterplots with linear regression line and shaded 95% confidence region for correlation estimation of *GSDME* transcript expression and EMT ssGSEA scores in CCLE lung cancer cell lines. R indicated Pearson correlation coefficient.

In line with our prior observation that GSDME was readily detectable in non-small cell lung cancer (NSCLC) (Lu et al., 2018), a panel of 30 cell lines exhibited a range of GSDME and GSDMD protein levels in immunoblotting analysis (Figure 1A). To assess whether GSDME abundance was regulated by gene methylation as reported, 12 models were selected and treated with two distinct DNA methyltransferase (DNMT) inhibitors, including a pan-DNMT inhibitor decitabine (Figure 1B) and a specific DNMT1 inhibitor thioguanine (Figure 1C). In a subset of NSCLC cell lines, GSDME expression, rather than GSDMD expression, was indeed restored by decitabine and thioguanine to various extent. In contrast, other NSCLC cell lines, e.g., NCI-H596, NCI-H358, and HCC4006, were not evidently impacted by either compound. Moreover, the promoter region of *GSDME* gene was mostly hypomethylated or partially methylated in cancer cell lines (Supplementary Figure S1A) and primary tumors (Supplementary Figure S1B). For comparison, the promoter region of *SOX10* gene was universally hypermethylated. These results suggested that *GSDME* gene methylation only partly accounted for its modulation and additional regulatory mechanisms might play an important role in human cancer.

We sought to systematically explore the biological determinants of GSDME levels in neoplastic cells. To this end, ssGSEA scores were calculated for a total of 9,243 hallmark gene sets and gene ontology terms (Subramanian et al., 2005) using CCLE RNA sequencing data (Ghandi et al., 2019), followed by correlation estimation with *GSDME* transcript quantity (Figure 1D). Interestingly, the analysis revealed that epithelial-mesenchymal transition was among the top hallmark pathways which were positively associated with *GSDME* expression (Figure 1E). The significant correlation was consistently observed with two different EMT-related transcriptional signatures in pan-cancer (Figure 1F) and lung cancer cell lines (Figure 1G). We further corroborated the finding of EMT-GSDME interconnection across 33 TCGA cancer types (Supplementary Figures S1C,D). Of note, the remaining five members of gasdermin family (GSDMA, GSDMB, GSDMC, GSDMD, and PJVK) did not show an obvious correlation with EMT ssGSEA scores in either cancer cell lines (Supplementary Figure S2) or primary tumors (Supplementary Figure S3). Together, our comprehensive investigations pinpointed that the EMT process might be specifically involved in GSDME regulation in lung and other human malignancies.

### Reversible GSDME Upregulation During Epithelial-Mesenchymal Transition

**FIGURE 2:**
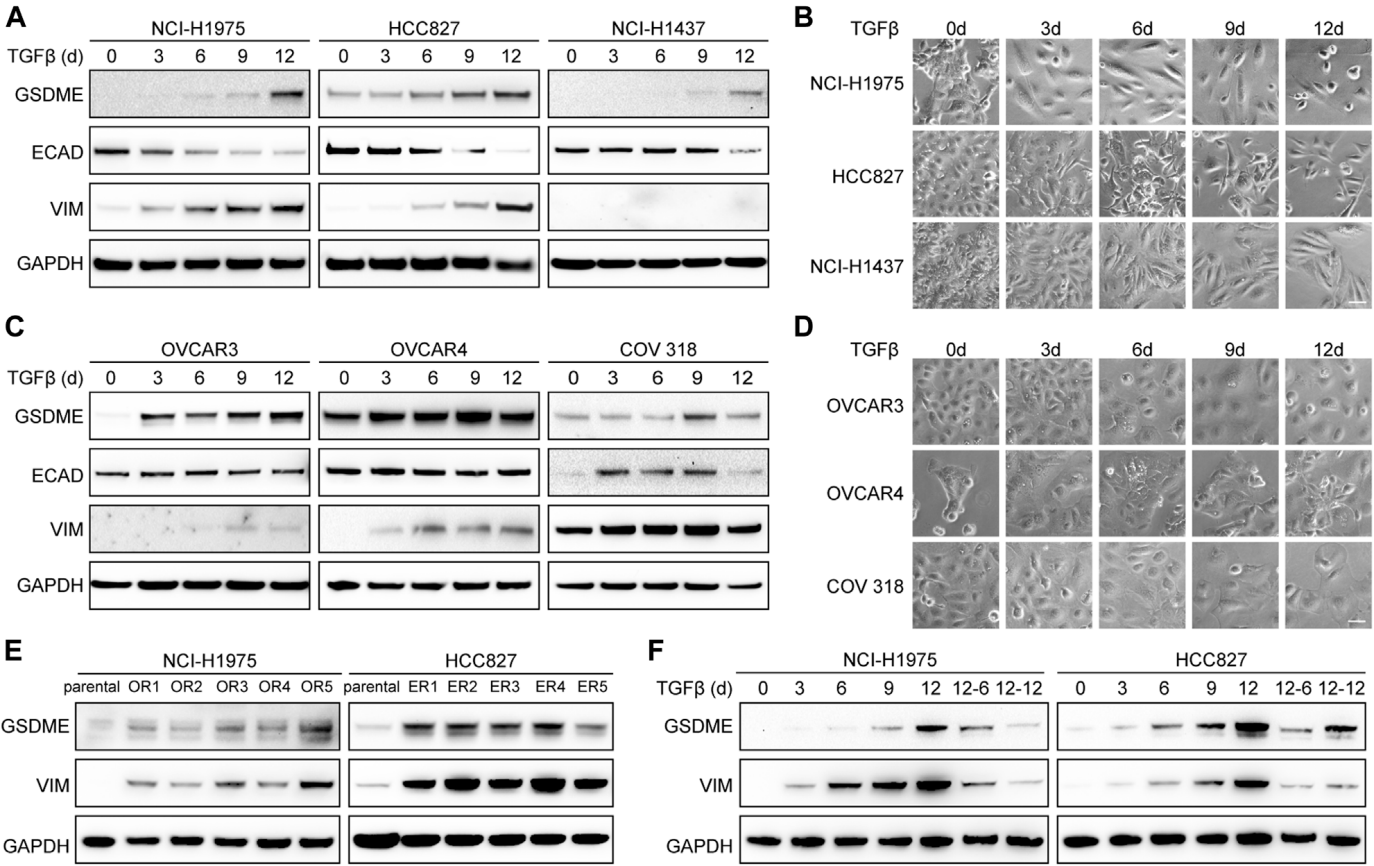
Reversible GSDME upregulation during EMT. **(A)** Three indicated lung cancer cell lines were treated with TGFβ (5 ng/μl) at a time course manner and analyzed by Western blot for GSDME protein expression. **(B)** Imaging analysis of lung cancer cells showed EMT morphology upon TGFβ treatment (scale bar = 50 μm). **(C)** Three indicated ovarian cancer cell lines were treated with TGFβ (5 ng/μl) at a time course manner and analyzed by Western blot for GSDME protein expression. **(D)** Imaging analysis of ovarian cancer cells showed EMT morphology upon TGFβ treatment (scale bar = 50 μm). **(E)** Osimertinib-resistant NCI-H1975 (OR) and erlotinib-resistant HCC827 (ER) cells were generated by long-term culture and analyzed by Western blot for GSDME protein expression. **(F)** NCI-H1975 and HCC827 cells were treated with TGFβ (5 ng/μl) to induce EMT. TGFβ was withdrawn as indicated and cells were analyzed by Western blot for GSDME protein expression.

To establish the causal role of EMT in GSDME upregulation, we first attempted to induce mesenchymal transdifferentiation with transforming growth factor-β (TGFβ) in a variety of cancer cell lines. As anticipated, elevated GSDME protein was detected at a time-dependent manner in lung cancer cells, such as NCI-H1975, HCC827, and NCI-H1437 (Figure 2A). Confirming previous reports (Miettinen et al., 1994; Zavadil and Böttinger, 2005; Thiery et al., 2009), TGFβ incited a gradual decrease of epithelial marker E-cadherin, a progressive increase of mesenchymal marker vimentin, and a corresponding acquisition of spindle-shaped morphology (Figure 2B). Similar GSDME upregulation along with EMT was noted in ovarian cancer cells including OVCAR3, OVCAR4, and COV 318 (Figures 2C,D). Furthermore, a larger panel of NSCLC cell lines verified that GSDME levels were augmented by TGFβ stimulation in most cases with just a few exceptions (Supplementary Figures S4A,B). In addition to TGFβ-mediated EMT, we introduced a distinct model (Sequist et al., 2011; Zhang et al., 2012) and validated GSDME upregulation by generating a serial of osimertinib-resistant NCI-H1975 and erlotinib-resistant HCC827 subclones (Figure 2E), which expectedly developed fibroblast-like features manifested as vimentin activation. Finally, upon TGFβ withdrawal from the NCI-H1975 and HCC827 mesenchymal derivatives, GSDME molecules demonstrated an analogous reversibility to the EMT program (Figure 2F). Therefore, EMT seemed to dictate GSDME expression in tumor cells.

### Direct Induction of *GSDME* Transcription by the Epithelial-Mesenchymal Transition Activators ZEB1/2

**FIGURE 3:**
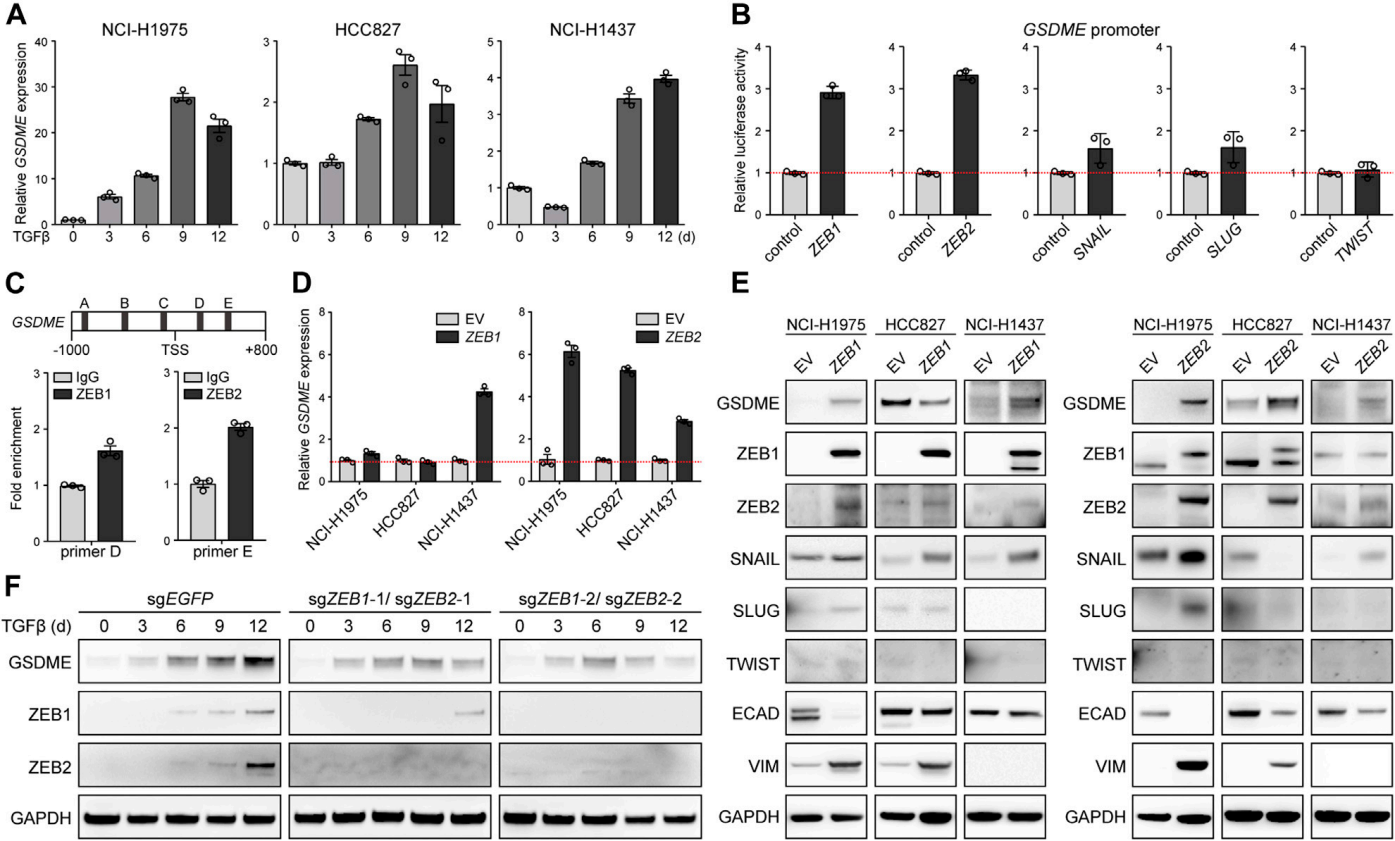
Direct induction of *GSDME* transcription by the EMT activator ZEB1/2. **(A)** Three indicated lung cancer cell lines were treated with TGFβ (5 ng/μl) at a time course manner and analyzed by qPCR for *GSDME* gene expression. Data are means ± SEM pooled from three independent experiments. **(B)** Five core EMT-TFs (*ZEB1*, *ZEB2*, *SNAIL*, *SLUG*, and *TWIST*) were co-transfected with *GSDME* promoter fused with dual luciferase reporter system and luciferase activity was assayed against the vector control. Data are means ± SEM pooled from three independent experiments. **(C)** *ZEB1* or *ZEB2* was overexpressed in NCI-H1975 cells and ChIP-qPCR analysis was performed with primer sets flanking five predicted ZEB1/2-binding consensus sequence proximal to the *GSDME* TSS (transcription start site). ZEB1 and ZEB2 was observed to bind to **(D,E)** locus at the *GSDME* promoter region, respectively. Data are means ± SEM pooled from three independent experiments. **(D)** EMT-TFs *ZEB1* or *ZEB2* was overexpressed in three indicated lung cancer cell lines and analyzed by qPCR for *GSDME* gene expression. Data are means ± SEM pooled from three independent experiments. **(E)** EMT-TFs *ZEB1* or *ZEB2* was overexpressed in three indicated lung cancer cell lines and analyzed by Western blot for GSDME protein expression. EV, empty vector. **(F)** *ZEB1* and *ZEB2* were simultaneously knocked out in HCC827 cells using the CRISPR-Cas9 system. Cells were treated with TGFβ (5 ng/μl) at a time course manner and analyzed by Western blot for GSDME protein expression.

We further dissected the molecular mechanism underlying EMT-associated GSDME regulation. Initially, quantitative PCR was performed in NCI-H1975, HCC827, and NCI-H1437 cells, and uncovered that *GSDME*, but not other gasdermins (Supplementary Figure S5A), was uniquely upregulated by TGFβ at the transcriptional level (Figure 3A). Likewise, five core EMT-inciting transcription factors (Stemmler et al., 2019), i.e., ZEB1, ZEB2, SNAIL, SLUG, and TWIST, displayed general elevation in response to TGFβ treatment (Supplementary Figure S5B), and thus were individually tested using a luciferase reporter fused with the *GSDME* promoter. Remarkably, overexpressed ZEB1/2 significantly enhanced the luciferase activity (Figure 3B) and consistently, endogenous ZEB1/2 directly bound to a consensus motif located in the *GSDME* promoter (Figure 3C), implying a crucial role of the classical ZEB family EMT-TFs. Indeed, ZEB1 and ZEB2 exhibited positive correlation with *GSDME* transcript across 188 CCLE lung cancer cell lines (Supplementary Figure S6A). Constitutive ectopic expression of ZEB1/2 (Supplementary Figure S6B) was competent to drive GSDME gene (Figure 3D) and protein upregulation (Figure 3E). Notably, GSDME abundance was also increased in selected cell lines at the gene (Supplementary Figure S7A) and protein level (Supplementary Figure S7B) in the presence of exogenous SNAIL, SLUG or TWIST (Supplementary Figure S7C), implicating functional interplay and/or redundancy of different EMT-TFs. Nevertheless, synchronous *ZEB1* and *ZEB2* deficiency substantially attenuated TGFβ-conferred GSDME production in HCC827 cells (Figure 3F). These results collectively proved that core EMT-activating transcription factors, in particular ZEB1/2, were both sufficient and required for GSDME transcriptional induction.

### GSDME-Dependent Pyroptosis in Mesenchymal Cells Upon Drug Treatment

**FIGURE 4:**
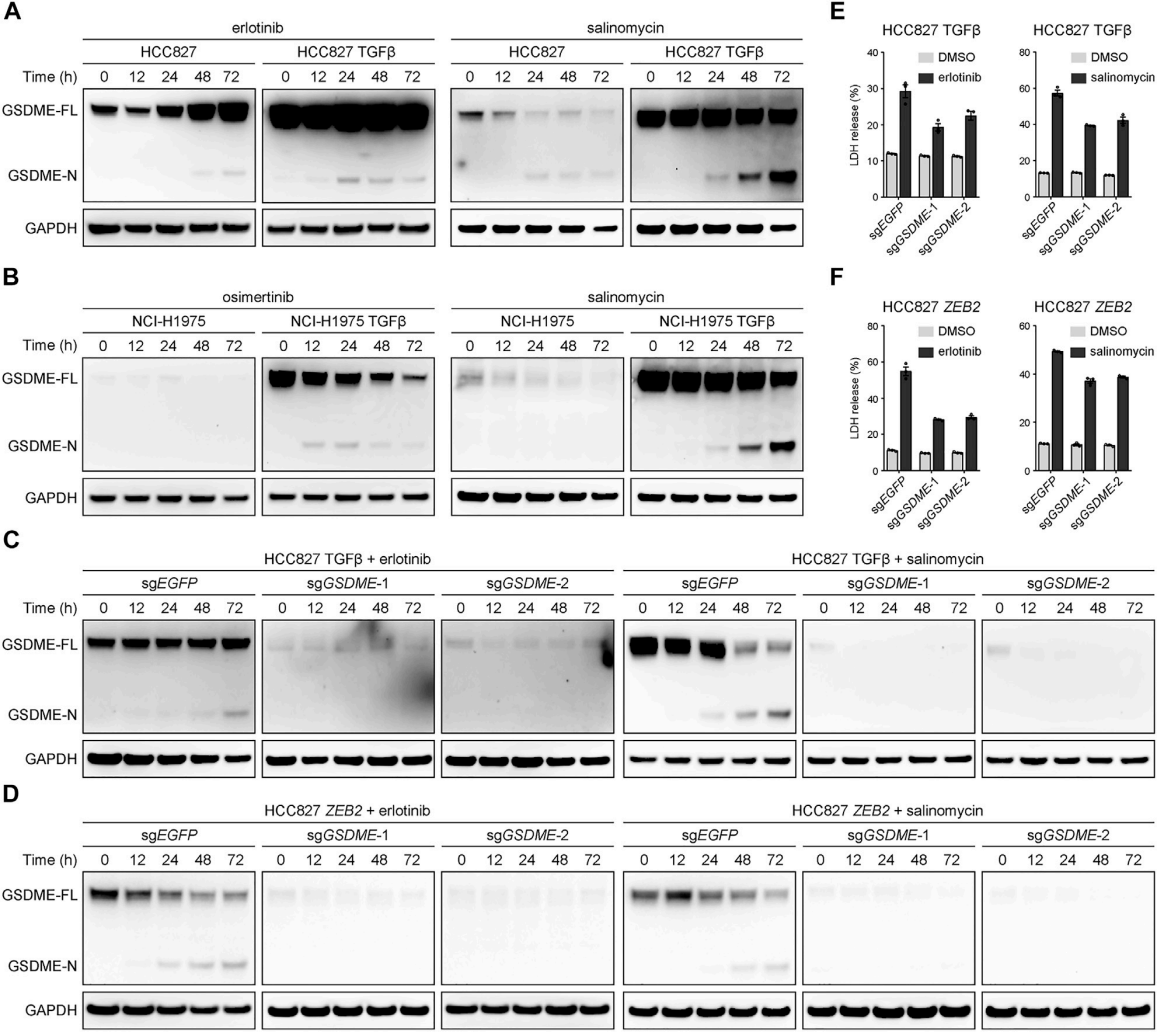
GSDME-dependent pyroptosis in mesenchymal cells upon drug treatment. **(A)** Parental and TGFβ-stimulated HCC827 cell lines were treated with erlotinib (4 μM) or salinomycin (4 μM) at a time course manner and analyzed by Western blot for GSDME protein cleavage. GSDME-FL, full-length GSDME; GSDME-N, GSDME N-terminal domain. **(B)** Parental and TGFβ-stimulated NCI-H1975 cell lines were treated with osimertinib (4 μM) or salinomycin (4 μM) at a time course manner and analyzed by Western blot for GSDME protein cleavage. **(C)** *GSDME* was knocked out in HCC827 cells using the CRISPR-Cas9 system. Cells were stimulated with TGFβ (5 ng/μl) to induce EMT, treated with erlotinib (4 μM) or salinomycin (4 μM), and analyzed by Western blot for GSDME protein cleavage. **(D)** *GSDME* was knocked out in HCC827 cells using the CRISPR-Cas9 system. Cells were transfected with exogenous *ZEB2* to induce EMT, treated with erlotinib (4 μM) or salinomycin (4 μM), and analyzed by Western blot for GSDME protein cleavage. **(E)** *GSDME* was knocked out in HCC827 cells using the CRISPR-Cas9 system. Cells were stimulated with TGFβ (5 ng/μl) to induce EMT, and LDH release in the presence of DMSO or indicated inhibitors was measured. Data are means ± SEM pooled from three independent experiments. **(F)** *GSDME* was knocked out in HCC827 cells using the CRISPR-Cas9 system. Cells were transfected with exogenous *ZEB2* to induce EMT, and LDH release in the presence of DMSO or indicated inhibitors was measured. Data are means ± SEM pooled from three independent experiments.

A key question emerged whether the elevated GSDME in mesenchymal cells underwent proteolytic cleavage to trigger cellular pyroptosis upon therapeutic treatment. To address this pivot biological relevance, alteration-matched EGFR tyrosine kinase inhibitors (erlotinib or osimertinib) and salinomycin, a potassium ionophore with EMT-specific toxicity (Gupta et al., 2009), were respectively assessed in parental and TGFβ-stimulated lung cancer models. In the mesenchymally transdifferentiated HCC827 (Figure 4A) and NCI-H1975 (Figure 4B) cell lines, these compounds invariably yielded more GSDME N-terminal fragments at a time-dependent manner, presumably unleashing its intrinsic pore-forming activity in response to drug exposure. Conversely, *GSDME* gene depletion using the CRISPR-Cas9 system was sufficient to eliminate therapy-induced GSDME cleavage products in TGFβ-mediated (Figure 4C) or *ZEB2*-overexpressing (Figure 4D) mesenchymal HCC827 cells. Consequently, *GSDME* knockout resulted in significantly decreased lactate dehydrogenase (LDH) release into the culture supernatant following erlotinib or salinomycin inhibition (Figures 4E,F). These data indicated that gained GSDME protein during EMT retained the capacity to execute cell-lytic pyroptosis.

### GSDME-Mediated Cytokine Release From Mesenchymal Cells

**FIGURE 5:**
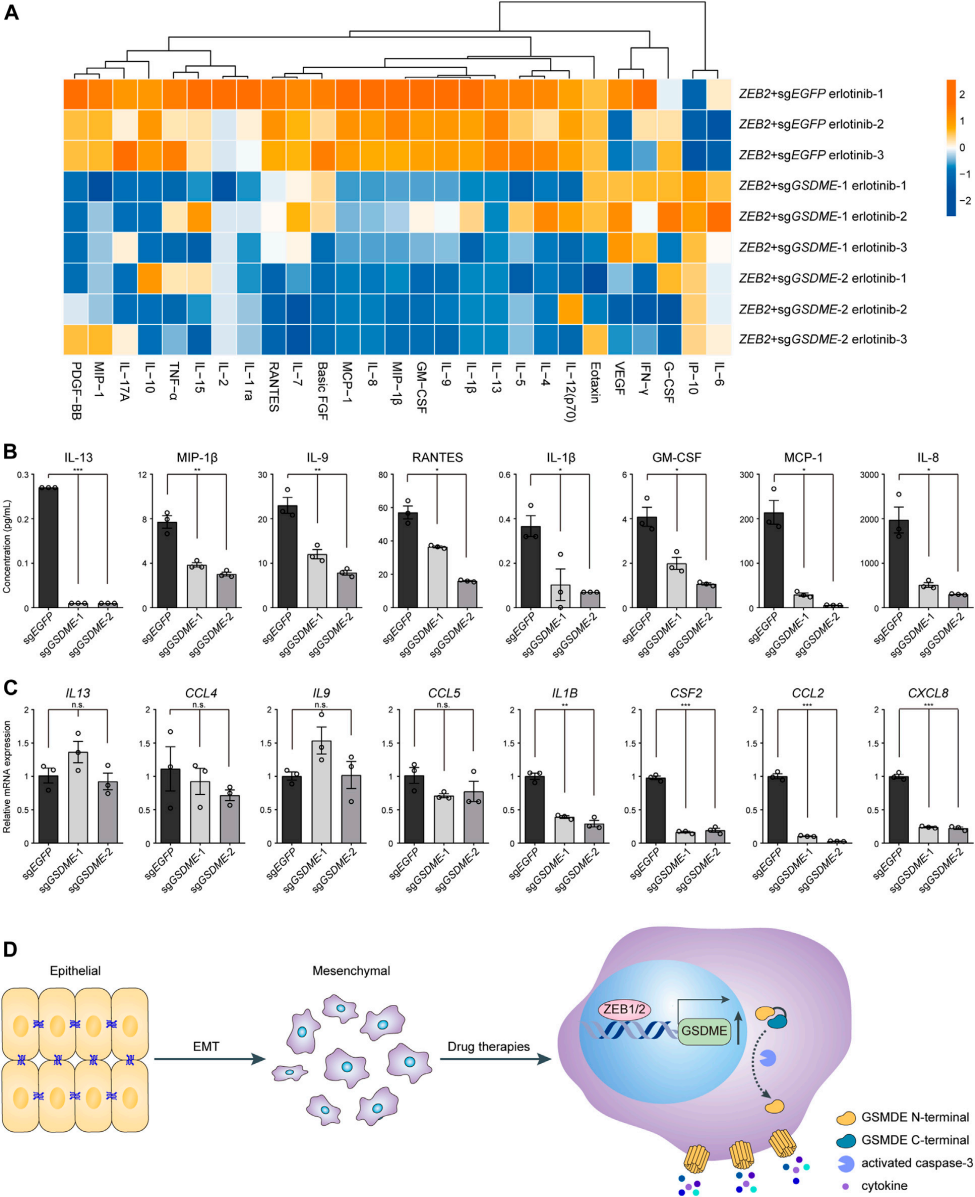
GSDME-mediated cytokine release from mesenchymal cells. **(A)** *GSDME* was knocked out in HCC827 cells using the CRISPR-Cas9 system. Cells were transfected with exogenous *ZEB2* to induce EMT, and treated with erlotinib (4 μM) for 72 h. The secretion of 27 cytokines was analyzed by Luminex liquid suspension chip. **(B)** Concentrations of eight representative cytokines as measured by Luminex liquid suspension chip (*p < 0.05; **p < 0.01; ***p < 0.001). Data are means ± SEM pooled from three independent experiments. **(C)** Gene expression levels of eight representative cytokines were analyzed by qPCR (n.s. not significant; *p < 0.05; **p < 0.01; ***p < 0.001). Data are means ± SEM pooled from three independent experiments. **(D)** A schematic summary of the study showing that EMT-TFs ZEB1 and ZEB2 induce *GSDME* transcriptional activation in mesenchymal tumor cells, leading to inflammatory pyroptosis with cytokine release in response to drug therapies.

Considering that pyroptosis represented a form of proinflammatory cell death, it was plausible to speculate that tumor immune milieu might be broadly altered by GSDME upregulation in transformed mesenchymal cells as compared to the epithelial counterparts. To experimentally test this hypothesis, Luminex liquid chip technology was adopted to probe a set of 27 common inflammatory and chemotactic cytokines in the conditional media of *ZEB2*-overexpressing HCC827 cells treated with erlotinib (Figure 5A). The multiplex assay was performed in triplicates and we found that the analyte concentrations were predominantly reduced following genetic *GSDME* ablation, as exemplified by eight representative cytokines displaying the most dramatic changes (Figure 5B). It was worth noting that gene expression at the mRNA level did not convincingly explain the observed differences of secreted proteins (Figure 5C). Based on these results, we concluded that GSDME-elicited pyroptosis would reshape the tumor immune attributes most likely by facilitating direct release of proinflammatory cytokines and potentially danger-associated molecular patterns in mesenchymally transdifferentiated cancers subjected to physiological stresses or therapeutic challenges.

## Discussion

While published work heavily focuses on the epigenetic modifications of *GSDME* gene, its transcriptional regulatory programs and their implications for therapeutic development remain understudied. Our findings build on prior literature and identify epithelial-mesenchymal transition with a previously unrecognized role in controlling GSDME expression. Mechanistically, core EMT-TFs ZEB1/2 bind to the *GSDME* promoter and directly drive gene transcription for the execution of pyroptotic cell death, leading to extracellular release of inflammatory cytokines (please refer to the schematic model in Figure 5D). These data shed light on the molecular determinants of GSDME abundance and predict unique susceptibility to GSDME-mediated pyroptosis for malignant cells of mesenchymal state or lineage.

Accumulative evidence highlights GSDME as a candidate tumor suppressor and a critical factor influencing treatment responses (Wang et al., 2017; Lu et al., 2018; Rogers et al., 2019). In addition, GSDME aggravates adverse effects of certain anticancer regimens by causing pyroptosis-based normal tissue damage or cytokine release syndrome (Huang et al., 2020; Liu et al., 2020). However, the intricate machinery governing its expression has not been fully elucidated, precluding further clinical translation to improve therapeutic efficacy and reduce therapy-related toxicity. In order to thoroughly understand the mechanisms underlying GSDME regulation, we performed integrative bioinformatics analyses on publicly available pan-cancer datasets and discovered that EMT gene signatures were among the top correlates of GSDME levels. Subsequently, the novel link between EMT and GSDME was experimentally confirmed with multiple models in different tumor types, exemplifying its validity and generalizability. Thus, as epithelial lesions acquire mesenchymal properties via EMT, a well-defined cellular process often triggered by microenvironmental signals, there may be a growing tendency for neoplasms to undergo pyroptotic rather than apoptotic cell death accordingly. Reinforcing this notion, our study also captured pronounced plasticity of GSDME-marked pathway during the reverse process, known as mesenchymal-epithelial transition (MET). It is important to mention the new view in the field that EMT does not act as binary switches and can be activated to various degrees (Grigore et al., 2016; Pastushenko et al., 2018; Yuan et al., 2019). Therefore, the modes and dynamics of GSDME modulation within a continuum of EMT intermediate states desire future investigations.

We found that core EMT-TFs ZEB1/2 were probably responsible for *GSDME* gene activation. The observation was reminiscent of previous findings on other gasdermin members illustrating molecular regulation at the transcriptional level. For example, transcription factor IRF2 was identified as a direct driver of *GSDMD* expression in a spectrum of cell types (Kayagaki et al., 2019), and *GSDMC* transcription was enhanced by nuclear PD-L1/p-STAT3 co-activators under hypoxia (Hou et al., 2020). Despite that ZEB1 and ZEB2 normally serve as transcriptional suppressors of epithelial markers, they are able to exert pleiotropic functions depending on the cooperation with diverse co-factors (Zheng and Kang, 2014; Hegarty et al., 2015). Although we showed that ZEB1 and ZEB2 directly bound to the E-box motifs proximal to the *GSDME* transcription start site, whether ZEB1/2 acted individually or as a part of protein complexes to guide gene expression would require detailed exploration. In addition to transcriptional regulation, posttranslational mechanisms to fine-tune GSDME activity also exist and GSDME phosphorylation by an unknown kinase has been described to inhibit its pore-forming capacity (Rogers et al., 2019).

EMT has been linked to metastatic spread, cancer stemness and drug resistance, posing serious challenges to effective treatment (Thiery et al., 2009; Fischer et al., 2015; Zheng et al., 2015; Gainor et al., 2016; Shibue and Weinberg, 2017; Dongre and Weinberg, 2019; Williams et al., 2019). As such, our study promises to have profound therapeutic implications. First, because GSDME is upregulated along with the EMT process and remains functionally intact, pharmaceutical strategies activating GSDME-dependent pyroptosis present a specific opportunity for targeting human neoplasms with mesenchymal characteristics. Intriguingly, it is lately reported that high-mesenchymal state tumor cells, while often resistant to common therapeutics, are exquisitely vulnerable to ferroptosis, another lytic form of regulated cell death (Viswanathan et al., 2017; Sang et al., 2019). Hence, selected necrosis inducers can hold great potential for the inhibition of these typically unresponsive therapy-persisters (Hangauer et al., 2017). Second, pro-pyroptotic agents may simultaneously rejuvenate the immune microenvironment and render mesenchymally transdifferentiated cancers more susceptible to immunologic treatment by converting “cold” tumors into “hot” ones. Compared to the immunologically silent apoptosis, gasdermin-mediated pyroptosis is considered highly proinflammatory due to passive discharge of cytosolic contents that are enriched in cytokines and alarmins (Place and Kanneganti, 2019; Bedoui et al., 2020). Indeed, recent evidence has indicated that GSDME promotes lymphocyte infiltration and activation spontaneously or upon targeted inhibitors (Erkes et al., 2020; Wang et al., 2020; Zhang et al., 2020). In keeping with these reports, our data demonstrated that increased GSDME in mesenchymal cells facilitated drug-triggered cytokine release, though caution should be advised that some of them could also be detrimental to antitumor immunity. Continued fundamental research on GSDME regulation and biology to unravel its full immunostimulatory potential may ultimately improve clinical responses to current cancer immunotherapies.

## Data Availability

Publicly available datasets were analyzed in this study. This data can be found here: The Cancer Genome Atlas (TCGA) and Cancer Cell Line Encyclopedia (CCLE).
