# Supplementary material for: Epithelial-Mesenchymal Transition Induces GSDME Transcriptional Activation for Inflammatory Pyroptosis
Source: Front Cell Dev Biol. 2021 Nov 26;9:781365. doi: 10.3389/fcell.2021.781365 (PMC8660972; doi:10.3389/fcell.2021.781365)
Supplement: Supplementary file 1 [file DataSheet1.PDF]

Supplementary Figure 1

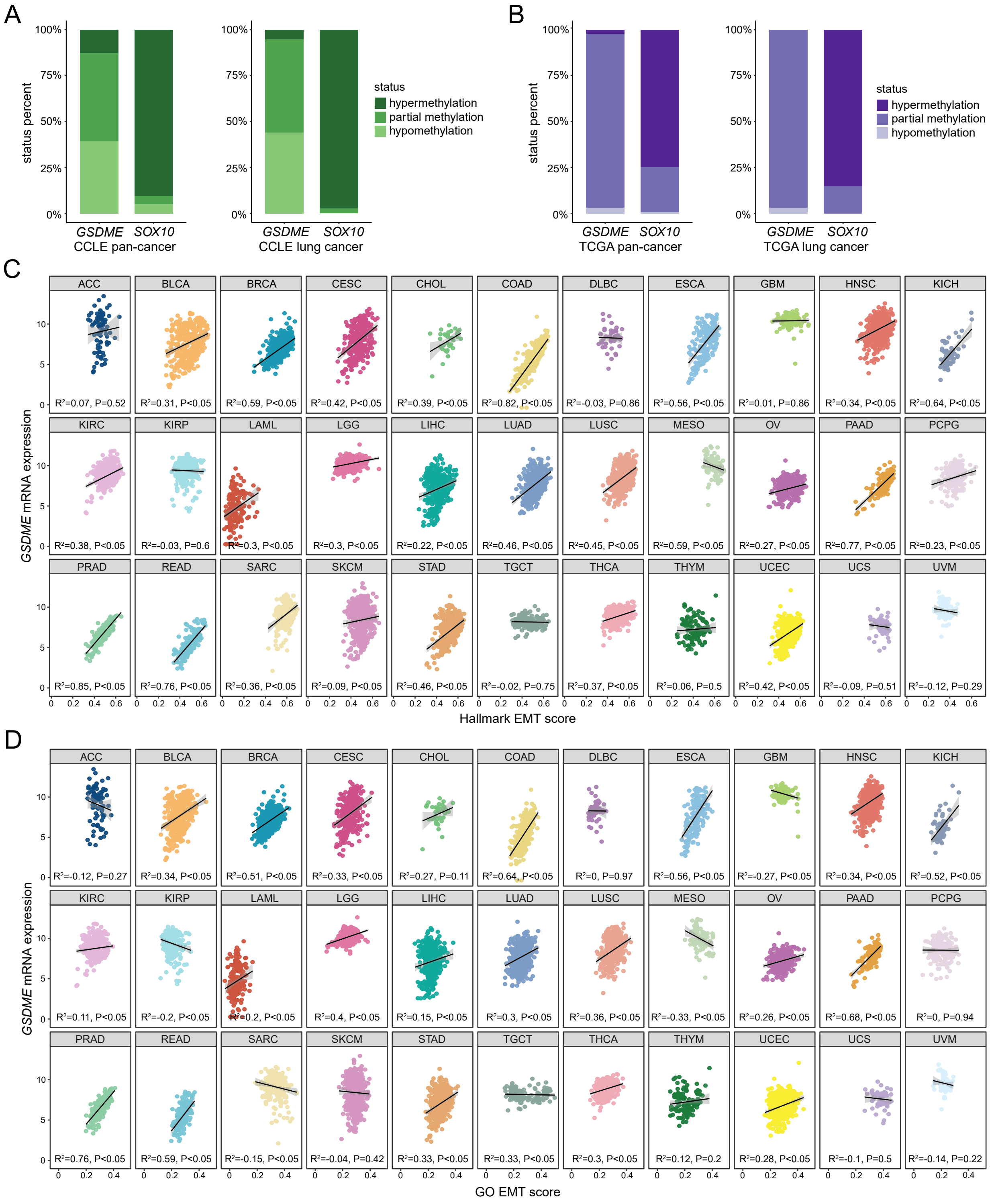

SFig 1. A. The methylation status of GSDME and SOX10 promoter region was analyzed in CCLE pan-cancer and lung cancer cell lines. B. The methylation status of GSDME and SOX10 promoter region was analyzed in TCGA pan-cancer and lung cancer samples. C. Scatterplots with linear regression line and shaded 95% confidence region for correlation estimation of GSDME transcript expression and hallmark EMT ssGSEA scores across 33 TCGA cancer types. R indicated Pearson correlation coefficient. D. Scatterplots with linear regression line and shaded 95% confidence region for correlation estimation of GSDME transcript expression and gene ontology EMT ssGSEA scores across 33 TCGA cancer types. R indicated Pearson correlation coefficient.

Supplementary Figure 2

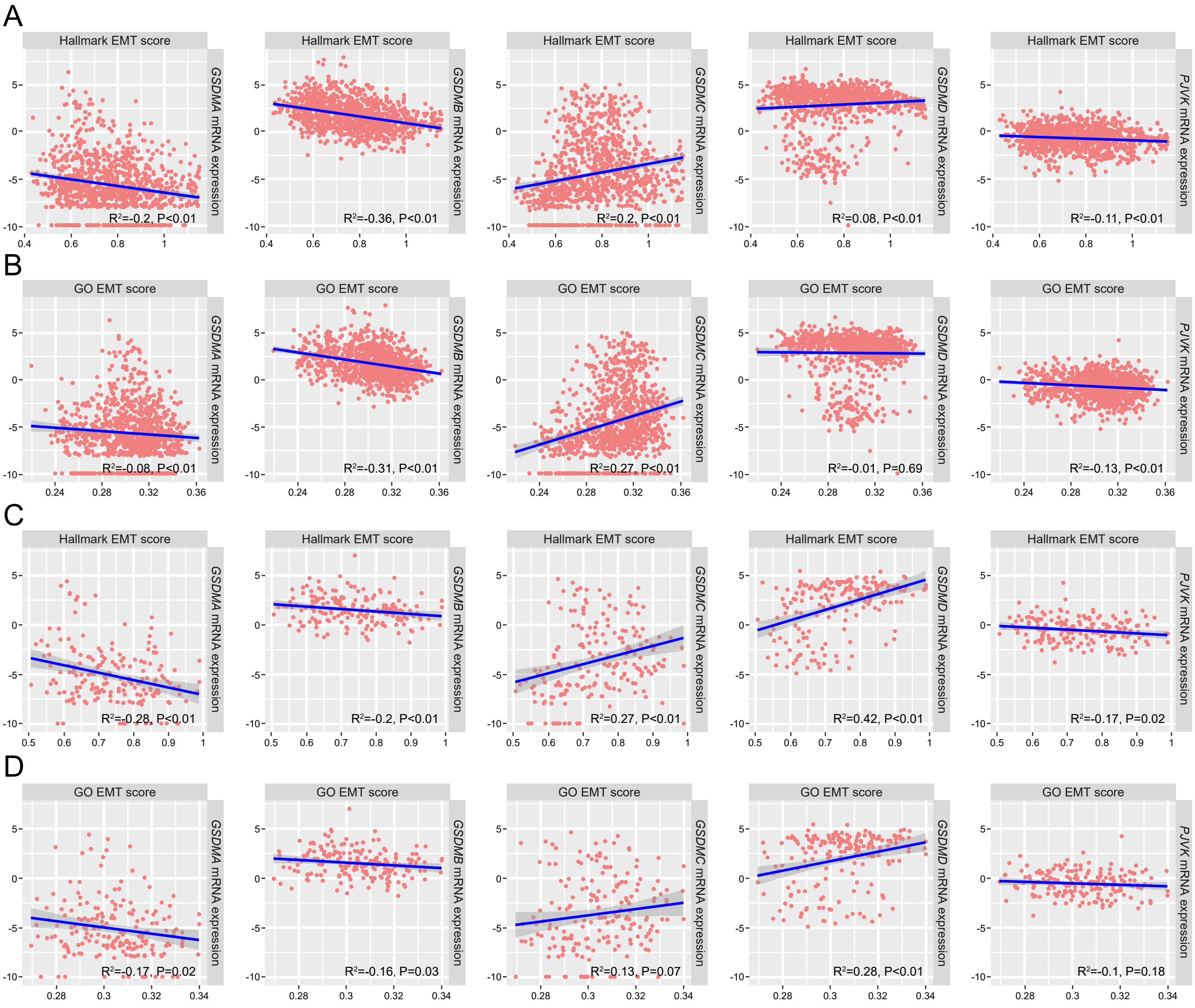

SFig 2. A. Scatterplots with linear regression line and shaded 95% confidence region for correlation estimation of other gasdermin transcript expression and hallmark EMT ssGSEA scores in CCLE cancer cell lines. R indicated Pearson correlation coefficient. B. Scatterplots with linear regression line and shaded 95% confidence region for correlation estimation of other gasdermin transcript expression and gene ontology EMT ssGSEA scores in CCLE cancer cell lines. R indicated Pearson correlation coefficient. C. Scatterplots with linear regression line and shaded 95% confidence region for correlation estimation of other gasdermin transcript expression and hallmark EMT ssGSEA scores in CCLE lung cancer cell lines. R indicated Pearson correlation coefficient. D. Scatterplots with linear regression line and shaded 95% confidence region for correlation estimation of other gasdermin transcript expression and gene ontology EMT ssGSEA scores in CCLE lung cancer cell lines. R indicated Pearson correlation coefficient.

Supplementary Figure 3

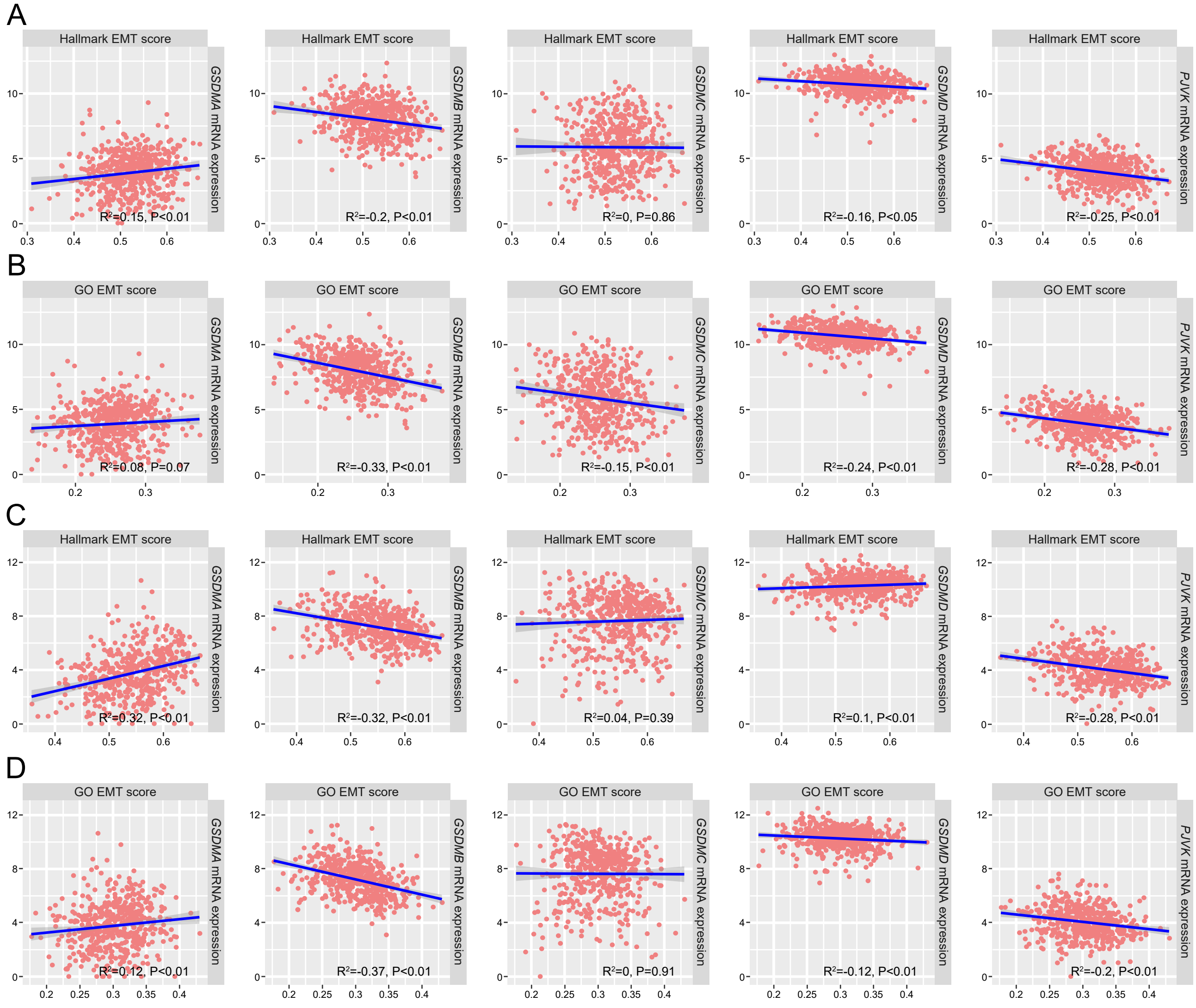

SFig 3. A. Scatterplots with linear regression line and shaded 95% confidence region for correlation estimation of other gasdermin transcript expression and hallmark EMT ssGSEA scores in TCGA lung adenocarcinoma. R indicated Pearson correlation coefficient. B. Scatterplots with linear regression line and shaded 95% confidence region for correlation estimation of other gasdermin transcript expression and gene ontology EMT ssGSEA scores in TCGA lung adenocarcinoma. R indicated Pearson correlation coefficient. C. Scatterplots with linear regression line and shaded 95% confidence region for correlation estimation of other gasdermin transcript expression and hallmark EMT ssGSEA scores in TCGA lung squamous cell carcinoma. R indicated Pearson correlation coefficient. D. Scatterplots with linear regression line and shaded 95% confidence region for correlation estimation of other gasdermin transcript expression and gene ontology EMT ssGSEA scores in TCGA lung squamous cell carcinoma. R indicated Pearson correlation coefficient.

Supplementary Figure 4

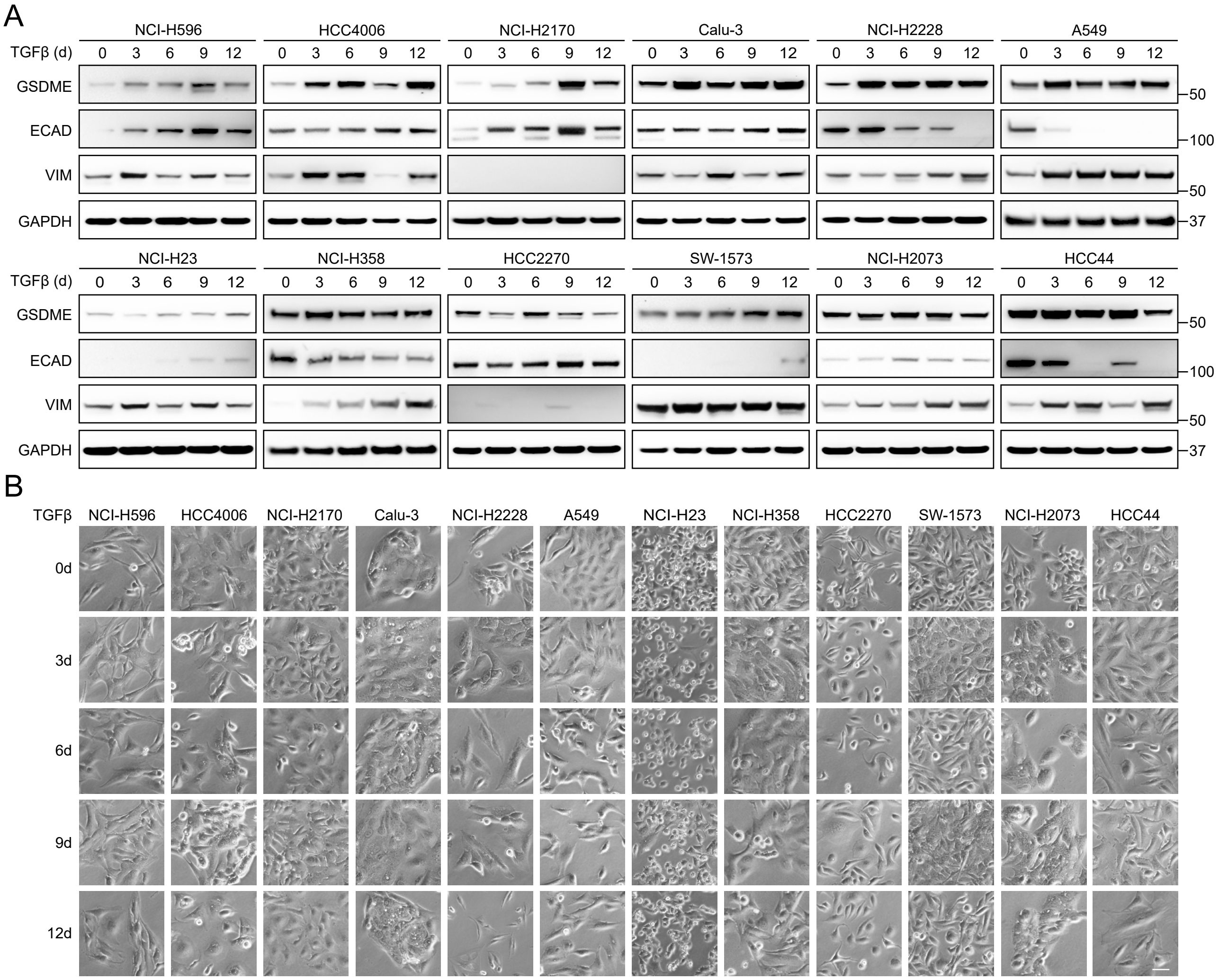

SFig 4. A. Indicated lung cancer cell lines were treated with TGFβ (5 ng/μL) at a time course manner and analyzed by Western blot for GSDME protein expression. B. Imaging analysis of indicated lung cancer cells showed EMT morphology upon TGFβ treatment (scale bar = 50 μm).

Supplementary Fig 5

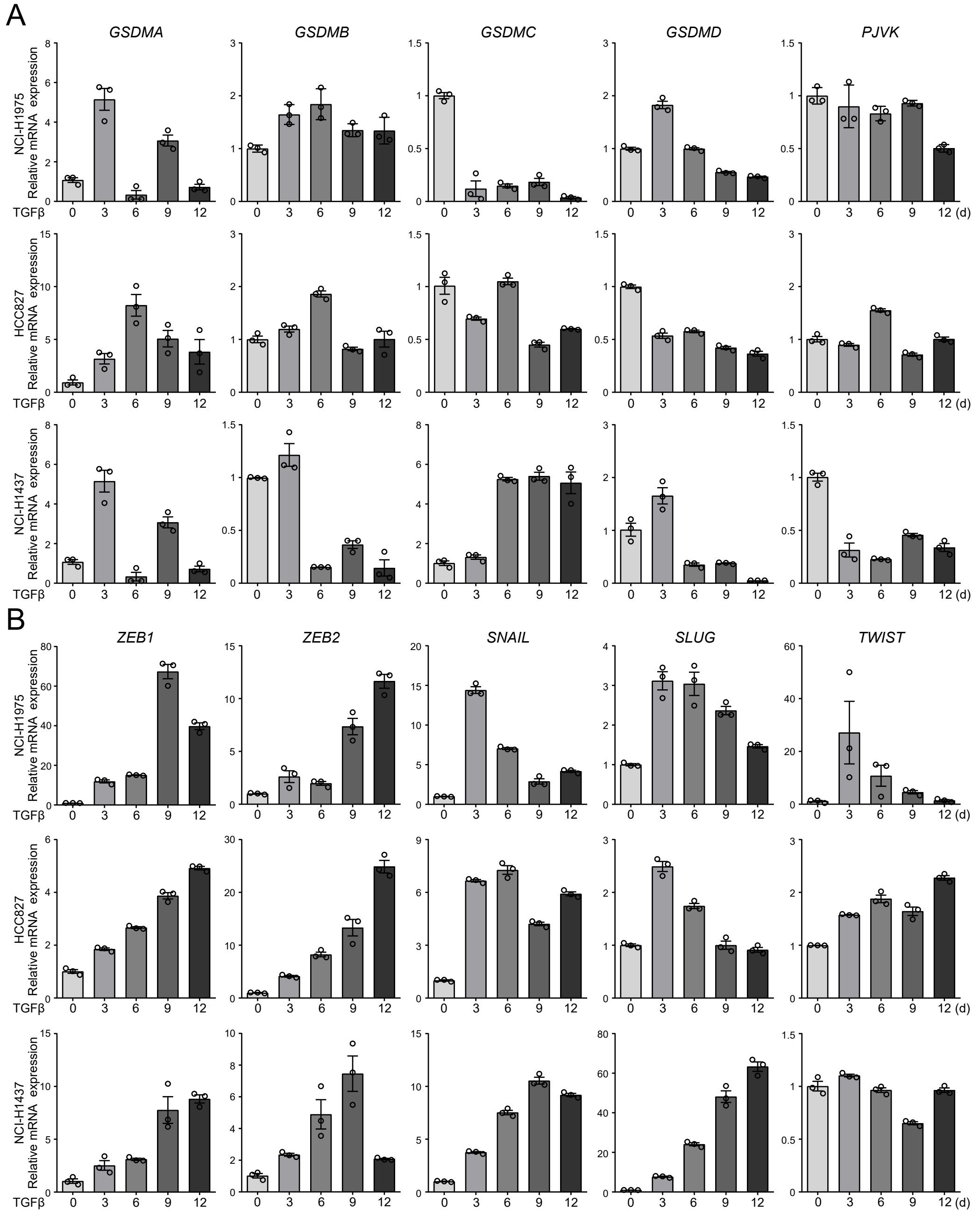

SFig 5. A. Three indicated lung cancer cell lines were treated with TGF-β (5 ng/μL) at a time course manner and analyzed by qPCR for other gasdermin gene expression. B. Three indicated lung cancer cell lines were treated with TGF-β (5 ng/μL) at a time course manner and analyzed by qPCR for gene expression of five core EMT-TFs. Data are means ± SEM pooled from three independent experiments.

Supplementary Fig 6

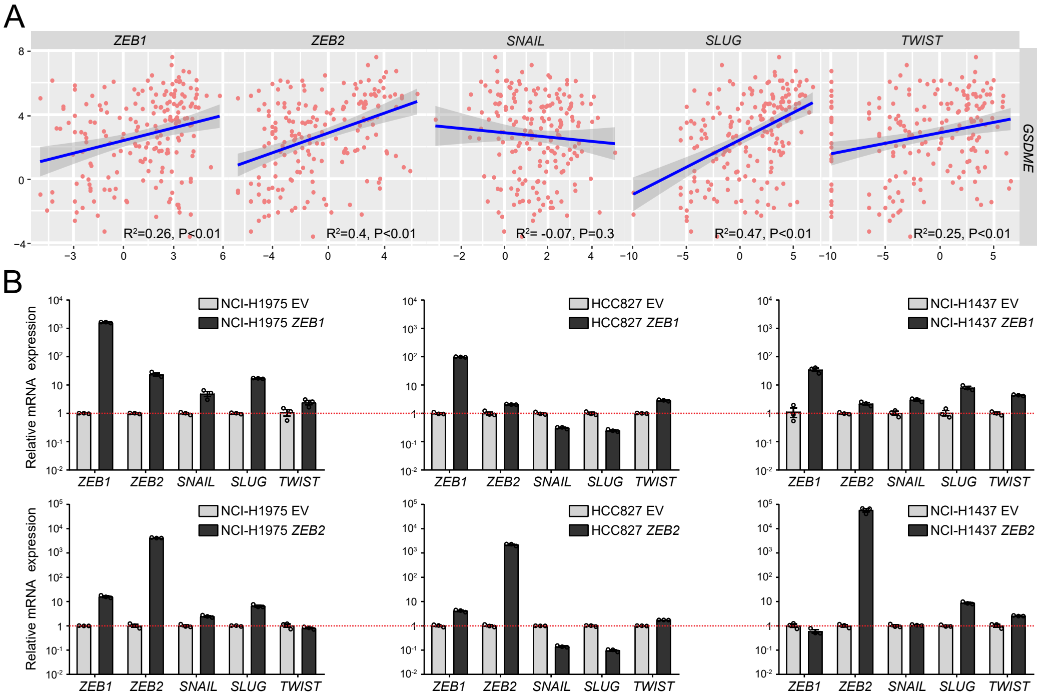

SFig 6. A. Scatterplots with linear regression line and shaded 95% confidence region for correlation estimation of transcript expression between GSDME and five core EMT-TFs in CCLE lung cancer cell lines. R indicated Pearson correlation coefficient. B. ZEB1 or ZEB2 was overexpressed in three indicated lung cancer cell lines and analyzed by qPCR for gene expression of five core EMT-TFs. EV, empty vector. Data are means ± SEM pooled from three independent experiments.

Supplementary Fig 7

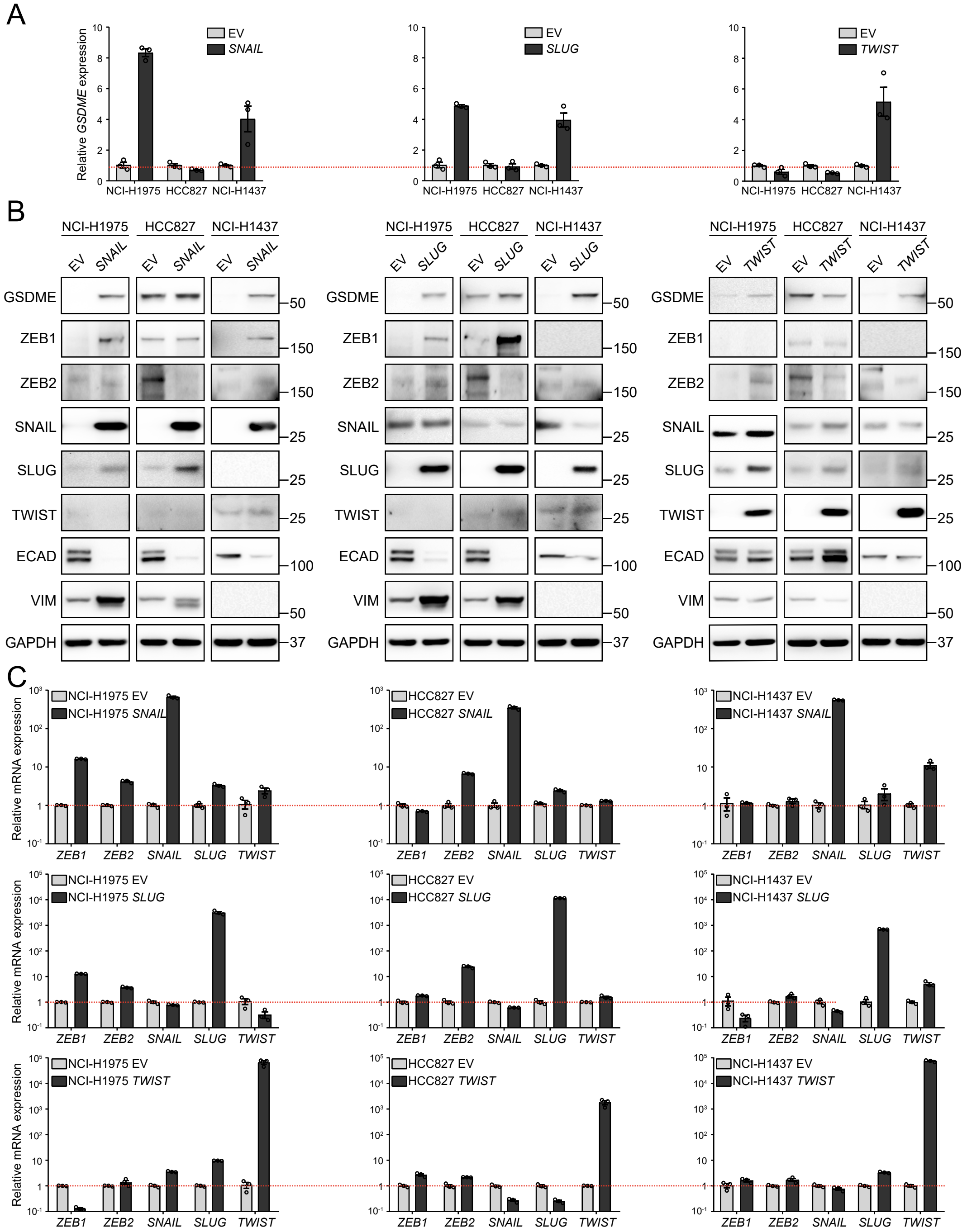

SFig 7. A. EMT-TFs SNAI1, SLUG or TWIST was overexpressed in three indicated lung cancer cell lines and analyzed by qPCR for GSDME gene expression. EV, empty vector. Data are means ± SEM pooled from three independent experiments. B. EMT-TFs SNAI1, SLUG or TWIST was overexpressed in three indicated lung cancer cell lines and analyzed by Western blot for GSDME protein expression. C. EMT-TFs SNAI1, SLUG or TWIST was overexpressed in three indicated lung cancer cell lines and analyzed by qPCR for gene expression of five core EMT-TFs. Data are means ± SEM pooled from three independent experiments.
